# Back pain as an initial feature of advanced gastric cancer mimicking multiple myeloma: A case report and literature review

**DOI:** 10.5339/qmj.2022.53

**Published:** 2022-11-09

**Authors:** Fateen Ata, Zohaib Yousaf, Bilal N. Al Kalaji, Anas A. Ashour, Mohamad Fael, Gi Eun Kim, Ammara Bint I Bilal, Orwa Elaiwy, Akhnuwkh Jones

**Affiliations:** ^1^Department of Internal Medicine, Hamad General Hospital, Hamad Medical Corporation, Doha, Qatar; ^2^Department of Radiology, Hamad General Hospital, Hamad Medical Corporation, Doha, Qatar; ^3^Department of Pathology, Hamad General Hospital, Hamad Medical Corporation, Doha, Qatar

**Keywords:** Back pain, Gastric cancer, Multiple Myeloma, Metastatic Malignancy

## Abstract

Background: Back pain is a rare initial presentation of gastric cancer. Isolated back pain with red flags in middle-aged patients might indicate multiple myeloma. However, it is rarely present in advanced gastric adenocarcinoma; hence, data are limited to case reports only. For a timely diagnosis of the underlying malignancy, endoscopy should be considered if the initial workup for this backache is unrevealing.

Case Presentation: We present a 34-year-old previously healthy gentleman with severe unremitting backache. He was ultimately diagnosed with gastric adenocarcinoma stage IV and received palliative treatment. The manuscript also reviewed relevant literature.

Conclusion: In rare cases, gastric malignancy can initially present as back pain with lytic bone lesions, mimicking multiple myeloma. Endoscopy early in the course of investigations may help reduce associated morbidities. Further, more extensive studies are required to understand better the clinical characteristics, demographics, and management of such patients.

## Background

Gastric cancer (GC) is a common malignancy. With more than 780,000 deaths annually, GC is the third deadliest malignancy among men.^1^ Majority of cases (approximately 75%) present with constitutional symptoms such as weight loss, appetite loss, and fatigue accompanied by specific symptoms such as hematemesis, melena, abdominal pain, vomiting, and fullness.^2^ GC can manifest atypically, leading to a diagnostic challenge and potential delay in timely diagnosis and management. Back pain accompanied by lytic lesions in the vertebrae is usually characteristic of multiple myeloma (MM).^3^ The incidence of bone metastasis from GC is estimated to be 13.4% in autopsy specimens.^4^ However, bone metastasis is higher in advanced stages than in early presentation.^5^ Back pain is sporadic as an initial complaint in patients diagnosed with GC, with a few cases reported in the literature.^6–20^ Identifying the etiology underlying such back pain is important. Differentiating the type of underlying malignancy based on the characteristics of the back pain, as in GC and MM secondary to bone metastasis causing a similar type of pain, is usually difficult. Signet cell carcinoma appears to be the most common histopathological type associated with back pain as an initial presentation in patients with GC.^7–9,11–13,19^ This report presents a similar case and reviews previously published cases to highlight this rare presentation of GC. In this case, the patient was male and presented with back pain, revealing lytic vertebral lesions that mimicked the common presentation of MM, ultimately revealing an advanced GC.

## Case Report

A 34-year-old Bangladeshi patient presented with severe back pain for 2 weeks. The low back pain was severe, which kept him up at night. He described it as the worst pain he had ever felt, characterizing it as a dull pain with 10/10 intensity. His pain was initially intermittent for the first few days but later became incessant, worsening with slight movement. The pain was non-radiating and did not tend to shift to any other parts of the body. However, it was associated with a different sharp, diffuse abdominal pain, which was persistent. He had two episodes of non-bilious, non-bloody vomiting the day before admission. He had no history of fever, weight loss, night sweats, melena, hematemesis, smoking, or alcohol use. He did not have any medical problems and was not taking any medications.

**Figure 1.**
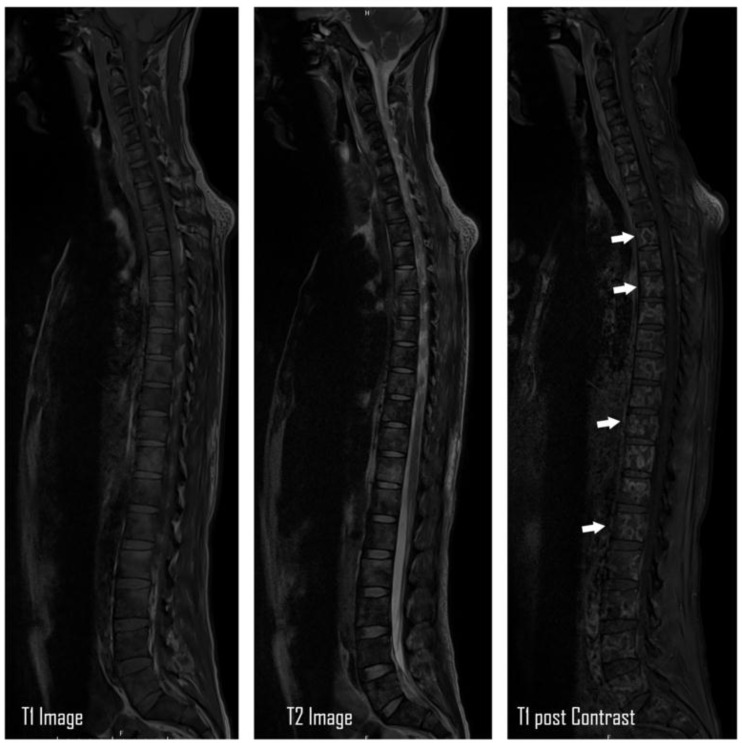
MRI spine showing multiple lytic lesions demonstrating post-contrast enhancement (widespread variable-sized bony metastatic versus myeloma lesions in the cervical, thoracic, lumbar, and sacral spine). Lesions exemplified with solid white arrows.

**Figure 2.**
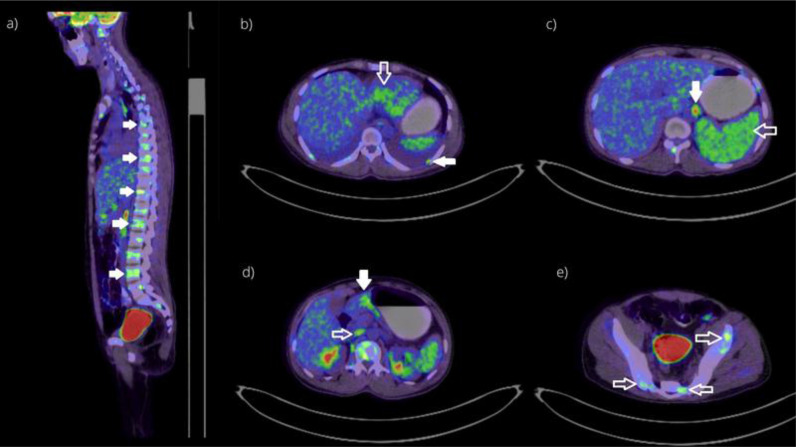
Positron emission tomography (PET scan) of the patient revealing multiple areas of uptake. (a) The solid white arrows show vertebral osteolytic lesions at multiple levels throughout the spine. (b) The solid white arrow denotes the uptake in left-sided pleural-based lesion, and the hollow white arrow shows the uptake in segment IV of the liver. (c) The solid white arrow demonstrates the uptake in the lateral aortic lymph node, and the hollow white arrow shows hypermetabolic spleen. (d) The solid white arrow shows the uptake in the pylorus of the stomach, and the hollow white arrow demonstrates the uptake in aortocaval lymph node. (e) The hollow white arrows denote lytic lesions in the bony pelvis.

The patient was afebrile (36.6°C) and had normal hemodynamic parameters (heart rate, 75 beats per minute; respiratory rate, 15 breaths per minute; oxygen saturation, 98% at room air). During the physical examination, back tenderness, abdominal tenderness, organomegaly, or palpable lymph nodes were not noted; only pallor was notable. Initial laboratory findings revealed microcytic anemia, thrombocytopenia, altered liver function tests with an obstructive pattern, and hyponatremia (Table 1). Further blood workup confirmed the presence of hemolytic anemia (with low haptoglobin, raised bilirubin, and high lactate dehydrogenase) (Table 1). Serum protein electrophoresis showed low albumin with an otherwise normal pattern. No monoclonal bands were detected. Urine protein electrophoresis showed a glomerular type of proteinuria. No band suggestive of free light chains was visualized, hence negative for Bence–Jones proteinuria. Computed tomography (CT) of the abdomen with oral and intravenous administration of contrast, conducted initially to rule out intestinal obstruction, incidentally showed suspicious thickening in the gastric pylorus and multiple osseous lytic lesions, which were later confirmed by magnetic resonance imaging (Figure 1). Given the initial presentation with multiple osteolytic lesions, hematologic malignancy was suspected, including MM. Positron emission tomography-CT showed increased uptake in multiple osseous lytic lesions, liver, retroperitoneal lymph nodes, and pleural lesions (Figure 2). However, serum electrophoresis and urine Bence–Jones proteins were negative.

**Figure 3.**
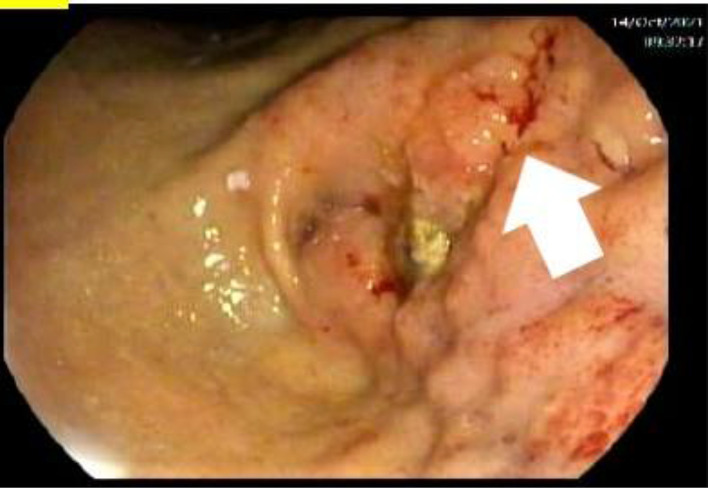
Endoscopy (solid white arrow) showing gastric mass (an irregular, infiltrative lesion causing partial obstruction of pyloric channel suggestive of adenocarcinoma or lymphoma)

**Figure 4.**
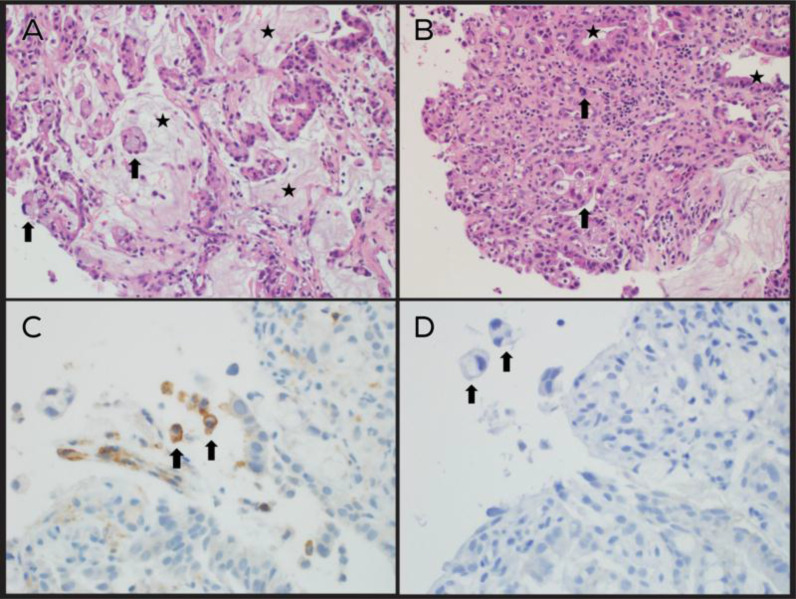
**(A)** Tumor cells aggregating in small clusters and single cells (black arrows) in pools of mucin (stars). Hematoxylin and eosin (H&E) staining, 200 × . **(B)** Tumor cells (black arrows) infiltrating gastric submucosa. Adjacent non-neoplastic mucosal glandular lining is also noted (stars). H&E staining, 200 × . **(C)** Tumor cells expressing PD-L1 immunostain (black arrows). PD-L1 immunohistochemical stain, 200 × . **(D)** Negative Her2/neu immunostain (black arrows point at tumor cells). Her2/neu immunohistochemical stain, 200 × .

Esophagogastroduodenoscopy, which was performed because of thickening in gastric pylorus on CT, showed an antral infiltrative lesion with partial pyloric channel obstruction, which suggested gastric adenocarcinoma or lymphoma (Figure 3). The gastric biopsy revealed a poorly differentiated adenocarcinoma with signet-ring cell and mucinous features and moderate chronic active gastritis associated with *Helicobacter pylori* (H. Pylori) (Figure 4). A diagnosis of stage IV GC (T4N3M1) was made. The cancer was poorly differentiated, Her-2 neu negative, and involved multiple regional lymph nodes. However, there was no peritoneal metastasis or ascites and no involvement of the liver.

A multidisciplinary team meeting was conducted, involving the internal medicine team, oncology team, hematology team, radiotherapist, histopathologist, gastroenterologist, and palliative team. Palliative care was decided due to the advanced stage of the disease. The patient received analgesics (morphine and gabapentin) for his worsening back pain, packed red blood cell transfusion, folic acid, and iron replacement. He also received the *H. pylori* eradication treatment.

Pyloric obstruction was stented. The patient was also treated with palliative radiotherapy to the lumbosacral spine (20 Gy in four fractions) and dexamethasone 4 mg intravenously twice daily to address his back pain and autoimmune hemolytic anemia, which was considered secondary to his malignancy. He completed the planned four radiotherapy sessions, and his back pain improved partially (enough to walk with assistance). The patient traveled back to his home country for further treatment.

## Discussion

GC is commonly asymptomatic and often presents in the advanced stages. Most commonly, GC presents as persistent abdominal pain associated with weight loss.^21^ Although abdominal pain presents in >60% of patients with GC, it is neither sensitive nor specific to GC.^22^ Less common symptoms present in 20%–35% include nausea, anorexia, dysphagia, and melena.^21^ We used PubMed, Scopus, and Google Scholar to conduct a systematic literature search using relevant search terms. We identified 610 articles, of which 10 were reviewed in detail after careful screening. We used the following MeSh terms to identify relevant articles from the databases: “stomach neoplasms” OR (“stomach” AND “neoplasms”) OR (“gastric” AND “cancer”) OR “gastric cancer” OR “adenocarcinoma” OR “adenocarcinomas” AND “back pain” OR (“back” AND “pain”) OR “vertebra.”*.”

Among the 10 patients discussed in this report (who presented with back pain and were eventually diagnosed with GC with metastatic spine lesions), 6 were men, and 4 were women. The average age was 51.2 (range, 35–76) years. More than 50% of the patients presented with lower back pain, whereas the rest had upper and mid back pain. Moreover, 30% of the patients reported anorexia and weight loss in addition to back pain. Fatigue and fever were concomitant symptoms in two patients. Results are summarized in Table 2.

Additionally, abdominal pain, bloating, heartburn, nausea, and vomiting were reported in 10% of the patients. None of the patients had hematemesis or melena. Most patients had no prior illness, except for one patient with hypertension and two patients with diabetes. Smoking was reported in 30% of the patients. Most of these patients were initially screened with X-ray imaging, but they all had further imaging with CT and MRI. Lytic and sclerotic lesions were equally predominant in radiological studies. Two patients had collapsed vertebrae. The most involved vertebrae were the thoracic and lumbar vertebrae. Only one patient had cervical spine involvement.

Interestingly, four patients were diagnosed by bone biopsy or bone scan, which warranted the gastric biopsy for definitive diagnosis. Gastric tumors were preponderant in the antrum. All patients had gastric adenocarcinoma, with 60% showing signet-ring cell features. Half of the patients were started on active treatment, whereas the rest received palliative care. The cancer progressed in 6 of the 10 patients, and they were deceased by the time their cases were reported.

While early satiety is a known symptom of GC, it presents in only 17% of patients and is often associated with later stages of the disease.^21^ Of these, weight loss, anorexia, dysphagia, and melena are considered alarm symptoms because they correlate with a worse prognosis.^23^ Stephens et al. followed 300 consecutive patients with gastric adenocarcinoma. They concluded that the presence of alarm symptoms correlate with a more advanced tumor stage and a more proximal location of the neoplasm.^24^ Another report of 4000 patients demonstrated that dyspepsia associated with only one of the alarm symptoms has a significant 26% reduction in 5-year survival rate compared with patients without alarm symptoms.^25^

Patients with GC with a long history of dyspepsia without associated alarm symptoms have less advanced stage and a better prognosis than patients with a short history of abdominal pain and alarm symptoms.^26^

Only a few cases of presentation with back pain from bone metastasis in GC has been reported, as presented above. It is essential to highlight this type of presentation, as it can mislead and direct the diagnostic search toward other diseases, which more commonly present in adulthood and early old age with back pain and lytic lesions in the vertebral column, especially MM.^3^ Hussain et al. reported a case where GC with early bone metastasis was complicated by bone marrow infiltration, causing anemia and disseminated intravascular coagulation.^27^ The coagulopathy also resulted in spinal hemorrhage and spinal cord compression.^27^ Sandilya et al. reported a patient with back pain associated with acute kidney injury who was found to have metastatic GC.^19^ However, the back pain was caused by peritoneal carcinomatosis and urinary bladder wall metastasis.^19^ Future studies are required to elucidate the behavior of GC metastasis and recommend specific treatment modalities to target such conditions.

The treatment goals of metastatic GC are prolongation of survival, improvement of the quality of life, and symptom palliation.^28^ The median survival ranges from 3 to 5 months with the best supportive care. In GC with bone metastasis, chemotherapy or radiation treatment reduce pain.^29^ Hironaka et al. reported the effectiveness of sequential methotrexate and 5-fluorouracil for GC with bone metastasis.^28^ In the 2008 S-1 plus cisplatin versus S-1 alone for first-line treatment of advanced GC trial, Koizumi et al. reported an increase in the median overall survival for S-1/cisplatin therapy (13 months) compared with S-1 alone (11 months).^2^ According to Yoshikawa and Kitaoka, radiation therapy was effective for the palliation of bone pain in 23 patients with GC associated with bone metastasis.^30^

## Conclusion

Back pain is a rare initial presentation of GC without accompanying signs or symptoms and appears to carry a poor prognosis. Clinicians must consider gastric malignancy while working up these patients for a diagnosis. Endoscopy should be a part of investigations with patients whose initial workup does not identify a cause. Further, more extensive studies are warranted to understand better the clinical characteristics, demographics, and management of such patients.

### Acknowledgment

None.

### Funding

This study was not funded.

### Conflict of interest

None of the authors have any conflict of interest to disclose.

### Ethics declaration

This work is original, has not been, and is not under consideration for publication in any other journal. All authors have reviewed and approved the final version of the manuscript. The study was approved by the Medical Research Centre (MRC) Qatar (MRC-04-21-912).

### Consent

Written informed consent for submitting this case report was provided by the patient

### Data sharing

Not applicable.

### Author contributions

FA: methodology, literature review, data collection and interpretation, manuscript writing, and finalization.

MB, AA, MF, and GK: literature review, data collection and interpretation, and relevant manuscript writing.

AB: provision of radiological images and relevant manuscript writing.

OE: provision of histopathological images and relevant manuscript writing.

ZY and AJ: supervision, literature review, and editing of the manuscript.

## Figures and Tables

**Table 1:** Relevant laboratory investigations of the patient during the hospital stay.

Investigations	Results	Reference range

Hgb	9.9 gm/dL	13–17 gm/dL

MCV	75.1 fL	83–101 fL

WBC	10.9 × 10^3^/μL	4–10 × 10^3^/μL

Platelets	113 × 10^3^/μL	150–400 × 10^3^μL

Retic %	2.3 %	0.5–2.5%

LDH	>1800 U/L	135–225 U/L

Haptoglobin	26 mg/dL	30–200 mg/dL

Prothrombin time	14.9 s	9.4–12.5 s

INR	1.3	

APTT	37.6 s	24.1–36.5 s

Serum sodium	127 mmol/L	136–145 mmol/L

Bilirubin T	32 μmol/L	0–21 μmol/L

Bilirubin D	17 μmol/L	0–5 μmol/L

ALT	75 U/L	0–41 U/L

AST	114 U/L	0–40 U/L

Alkaline phosphatase	478 U/L	40–129 U/L

CRP	149 mg/L	0–5 mg/L

Tumor markers		

AFP	3 IU/mL	0–6 IU/mL

CA 125	11.6 U/mL	0–35 U/mL

CA 15-3	22.7 U/mL	0–34.5 U/mL

CA 19-9	3.3 U/mL	0–27 U/mL

CEA	1421 μg/L	3.8–5.0 μg/L

Ka/La	1.26 ratio	0.26–1.65 ratio

KaFLC	23.5 mg/L	3.3–19.4 mg/L

LaFLC	18.6 mg/L	5.7–26.3 mg/L

24-h total volume	4200 mL	

24-h urine creatinine	8.43 mmol/24 h	9–21 mmol/24h

24-h urine protein	0.29 gm/24 h	0.03–0.15 gm/24hr

Urine osmolality	209 mmol/kg	150–1150 mmol/kg


AFP, alpha fetoprotein; CRP, C-reactive protein; ALT, alanine aminotransferase; APTT, activated partial thromboplastin time; AST, aspartate aminotransferase; CEA, carcinoembryonic antigen; Hgb, hemoglobin; INR, international normalized ratio; KaFLC, serum kappa free light-chain; LaFLC, serum lambda free light-chain; LDH, lactate dehydrogenase; MCV, mean corpuscular volume; WBC, white blood cell

**Table 2:** Clinical characteristics and outcomes of patients who presented with back pain and were diagnosed as gastric cancer.

Author, Year	Age (in years), sex	Reason for back pain	Diagnosis details	Level of vertebrae	Treatment of cancer	Outcome

Hussain, S., 2006 (17)	46, Female	Partial compression fracture	Adenocarcinoma Poorly differentiated adenocarcinoma of diffuse type	T2, tbl10, tbl12, L3, and L5	Palliative	Died

Sandilya, S., 2011 (19)	48, Male	Bilateral ureteral obstruction	Signet-ring cell carcinoma Fibroadipose tissue with infiltrating poorly differentiated adenocarcinoma with signet-ring cell features	Not mentioned	Palliative	Died

Basheer, A., 2013 (7)	35, Male	Lytic and blastic lesions Collapsed vertebrae	Adenocarcinoma Poorly differentiated adenocarcinoma	C5, C6, tbl8, tbl11, and L2	Palliative	Alive

Dittus, C., 2014 (11)	52, Male	Lytic lesions	Signet-ring cell carcinoma Positive staining of signet-ring cells on bone marrow biopsy	T5 and tbl9	Active	Alive

Ameur, W. B, 2017 (6)	Middle- aged, Female	Lytic lesions	Adenocarcinoma Moderately differentiated and infiltrating of the stomach	T5, tbl9, and tbl11	Active	Alive

Becerra-Pedraza, L. C., 2017 (8)	47, Male	Lytic lesions	Signet-ring cell carcinoma Cellular atypia and pleomorphic cells infiltrated in all the thickness of the gastric wall	T11– L4	Palliative	Died

Fan, P, 2017 (12)	41, Female	Blastic lesions	Signet-ring cell carcinoma Undifferentiated adenocarcinoma on bone marrow biopsy Gastric biopsy showed signet-ring cell carcinoma	Widespread	Declined treatment	Died

Sarah Burroughs., 2020 (9)	53, Male	Blastic lesions	Signet-ring cell carcinoma Extensive infiltration by sheets of malignant epithelial cells	L2, L3	Active	Died

Chitty, A., 2020 (10)	76, Female	Not mentioned	Adenocarcinoma Infiltrating, poorly differentiated adenocarcinoma	Not mentioned	Active	Alive

Fujita, I., 2020 (13)	63, Male	Not mentioned	Signet-ring cell carcinoma Gastric biopsy demonstrated a signet-ring cell carcinoma Bone biopsy revealed cell aggregation, indicating signet-ring cell carcinoma	Thoracic and lumbar	Active	Died

